# Supplementary material for: PD-1/PD-L1 checkpoint blockade harnesses monocyte-derived macrophages to combat cognitive impairment in a tauopathy mouse model
Source: Nat Commun. 2019 Jan 28;10:465. doi: 10.1038/s41467-019-08352-5 (PMC6349941; doi:10.1038/s41467-019-08352-5)
Supplement: Supplementary file 1 — Supplementary Information [file 41467_2019_8352_MOESM1_ESM.pdf]

Supplementary Fig. 1. A single injection of 0.5mg anti–PD-1 in 5XFAD female mice has a similar beneficial effect on cognitive performance, as two injections of 0.25mg.

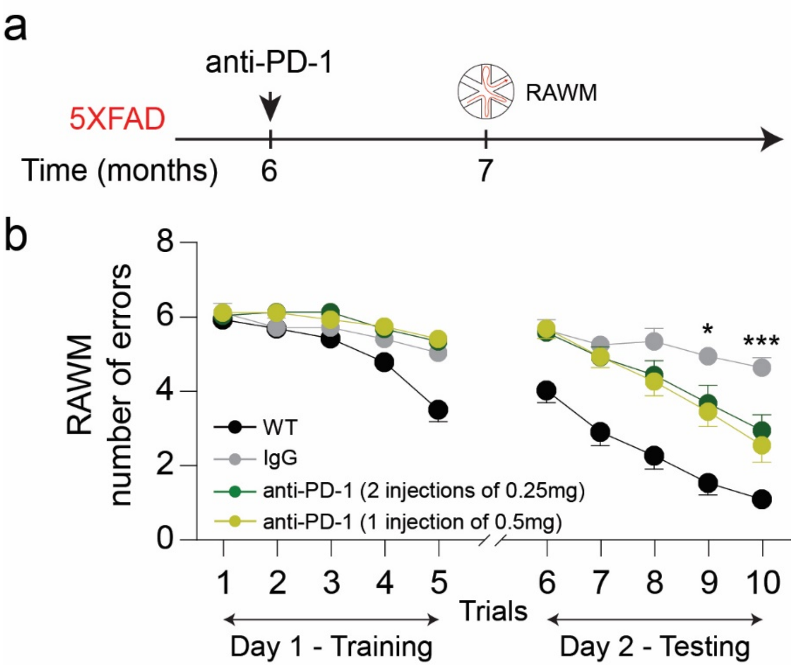

Supplementary Fig. 1. A single injection of 0.5mg of anti–PD-1 in 5XFAD female mice has a similar beneficial effect on cognitive performance, as two injections of 0.25mg. Female 5XFAD mice (average age 7 months) were treated with either two injections of 0.25mg anti-PD-1 or with a single injection of 0.5mg PD-1–specific antibody or isotype matched control antibody (IgG). Experimental design is presented in (**a**). Black arrow indicates time point of treatment, and illustrations indicate time points of cognitive scoring using the Radial arm water maze (RAWM). (**b**) RAWM performance of 5XFAD mice treated with two injections of 0.25mg of anti–PD-1 (n = 11), 5XFAD mice treated with a single injection of 0.5mg of anti–PD-1 (n = 7), 5XFAD mice treated with IgG isotype control (n = 10), and wild-type (WT) (n = 14) controls. Two-way repeated-measures ANOVA and Dunnett's post-hoc test for multiple comparisons. Data are represented as mean ± s.e.m.; $^{*}P<0.05$, $^{**}P<0.01$, $^{***}P<0.001$.

Supplementary Fig. 2. Early treatment with anti-PD-1 antibody rescues neurons in 5XFAD mice.

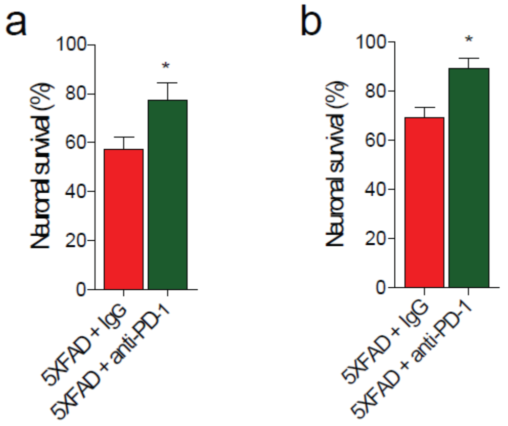

Supplementary Fig. 2. Early treatment with anti-PD-1 antibody rescues neurons in 5XFAD mice.

Repeated analysis of neuronal survival of the experiment described in Figure 1 a-d, assessed in the subiculum by Cresyl violet-staining (**a**), and by Neu-N^{+} immune-reactivity (**b**), in which we included animals that had a motor deficit in all groups, and could not perform the cognitive test that required swimming. Anti–PD-1–treated 5XFAD mice (n = 9), and IgG-treated 5XFAD mice (n = 10), respectively; Results are expressed as percentage of surviving neurons relative to the number of the pyramidal neurons in the age-matched the WT littermates (n = 6) controls, which were also analyzed. Data are represented as mean ± s.e.m.; *P<0.05, **P<0.01, ***P<0.001.

Supplementary Fig. 3. Blockade of PD-L1 reduces cerebral pathology in 5XFAD mice.

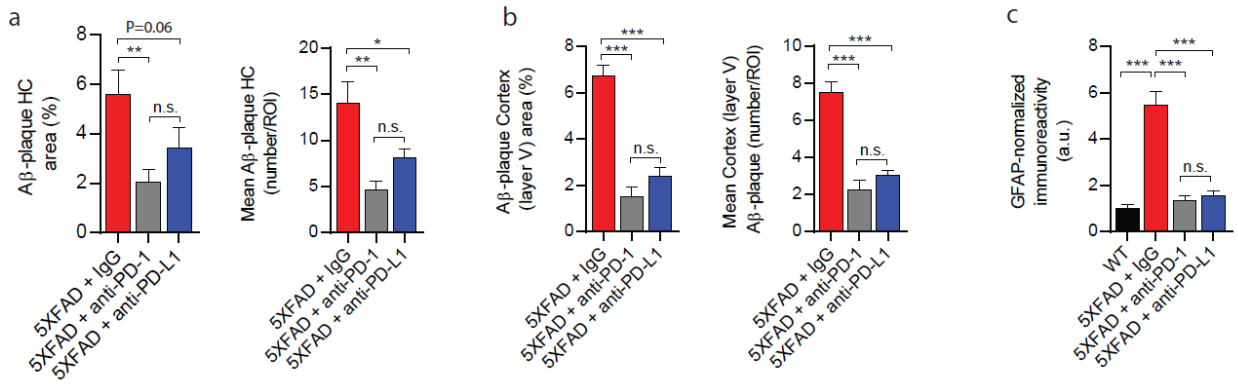

Supplementary Fig. 3. Blockade of PD-L1 reduces cerebral pathology in 5XFAD mice. Immunohistochemical analysis of the mice described in Figure 2d-g, including those that were excluded due to motor deficits in the behavior (Fig. 2c) and the initial pathological analyses (Fig. 2e-g). (**a, b**) quantification of Aβ immunoreactivity in anti–PD-1–treated 5XFAD mice (n = 9), anti–PD-L1-treated 5XFAD mice (n = 10), and IgG-treated (n = 9) 5XFAD mice. (**c**) GFAP in anti–PD-1–treated (n = 8), anti–PD-L1-treated 5XFAD mice (n = 10), IgG-treated (n = 15) 5XFAD mice, and WT (n=6), assessed 1 month after treatment. Mean plaque area and plaque numbers were quantified in 6-µm brain slices in the dentate gyrus (DG) and in the cerebral cortex (layer V), and GFAP immunoreactivity was measured in the hippocampus (one-way ANOVA and Fisher's exact test).

Supplementary Figure 4. Separate analysis of the effect of IgG2a and IgG2b on behavior in DM-hTAU mice

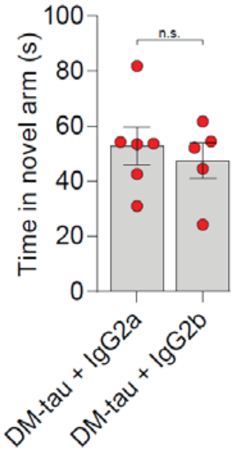

Supplementary Figure 4. Separate analysis of the effect of IgG2a and IgG2b on behavior in DM-hTAU mice. Separate analysis of DM-hTAU mice, which were treated with either IgG2a (n=6) or IgG2b (n=5), tested in T-maze task, and analyzed together in Figure 3c. The two IgG isotypes controls showed a similar behavior, with no significant differences.

Supplementary Figure 5. Blockade of the PD-1/PD-L1 axis supports cognitive improvement in both male and female DM-hTAU mice.

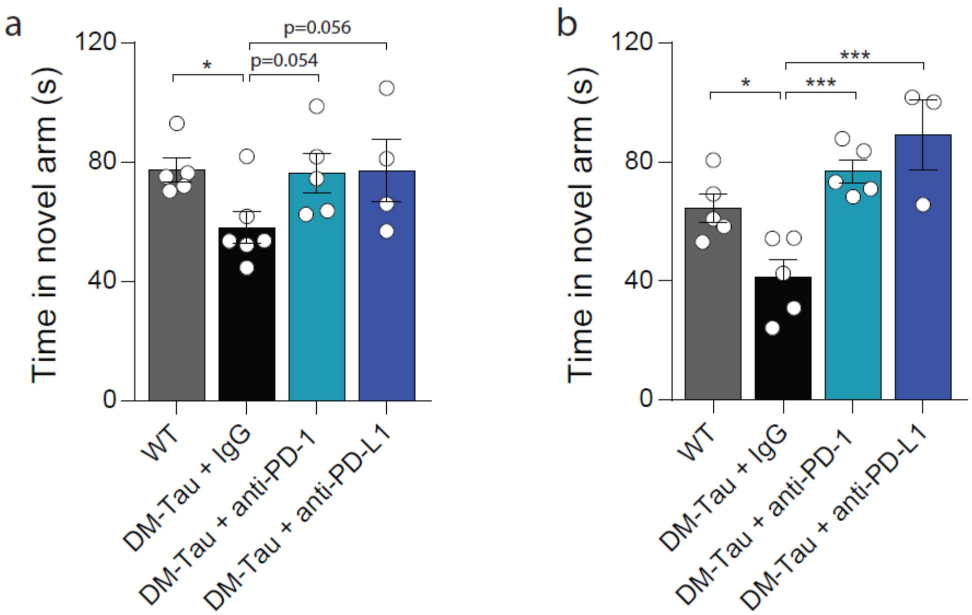

Supplementary Figure 5. Blockade of the PD-1/PD-L1 axis supports cognitive improvement in both male and female DM-hTAU mice. Male and female mice expressing the human-tau gene with two mutations (K257T/P301S; double mutant, DM-hTAU) (average cohorts aged 8 months) were treated with anti-PD-1–specific antibody, anti-PD-L1–specific antibody, or isotype matched control antibody (IgG) (one i.p. injection of 0.5mg/mouse); experimental design is presented in Fig. 3a. The results shown here are of seperately analyzing the the male (a) and female (b) in Figure 3c. (**a**). Effect of PD-1/PD-L1 blockade on spatial memory using T maze task. DM-hTAU male mice treated with either anti–PD-1 (n = 6) or anti–PD-L1(n = 4) exhibited preference for the novel arm relative to IgG controls (n=6); age matched wild-type (WT) littermates (n=5) were used as an additional control group. (**b**) Effect of PD-1/PD-L1 blockade on spatial memory using T maze task. DM-hTAU female mice treated with either anti–PD-1 (n = 4) or anti–PD-L1(n = 3) exhibited preference for the novel arm relative to IgG controls (n=5); age matched wild-type (WT) littermates (n=5) were used as an additional control group.

Supplementary Figure 6. Blockade of the PD-L1 in WT does not affect cognitive performance.

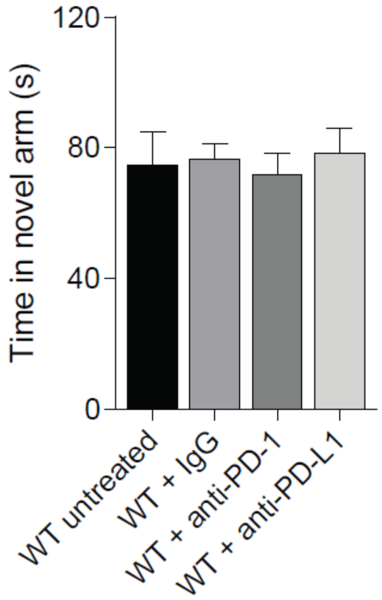

Supplementary Figure 6. Blockade of the PD-L1 in WT does not affect cognitive performance. Male WT littermates from the DM-hTAU colony (average cohorts aged 8 months) were treated with anti-PD-L1–specific antibody, or isotype matched control antibody (IgG) (one i.p. injection of 1.5mg/mouse) and tested for cognitive performance using T maze. WT littermates treated with anti-PD-1 (n=5) or with anti-PD-L1 (n=4) performed similarly to IgG control treated mice (n=5). Untreated WT littermates (n=5) were used as an additional control group.

Supplementary Figure 7. Blockade of PD-L1 rescues cognitive performance in DM-hTAU mice.

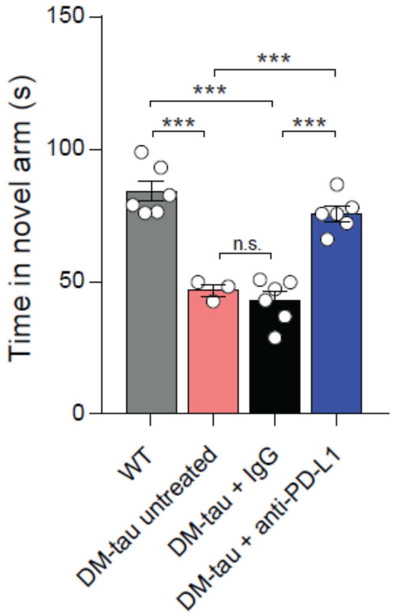

Supplementary Figure 7. Blockade of PD-L1 rescues cognitive performance in DM-hTAU mice. Male and female mice DM-hTAU mice (average cohorts aged 8 months) were treated with anti-PD-L1 (n=6) or with IgG control (n=6) (one i.p. injection of 1.5mg/mouse) and compared to untreated DM-hTAU littermates (n=3), using the T maze task 1 month after the treatment. WT littermates (n=6) were used as an additional control group.

Supplementary Figure 8. Single cell RNA-seq reveals heterogenous population of infiltrating monocytes.

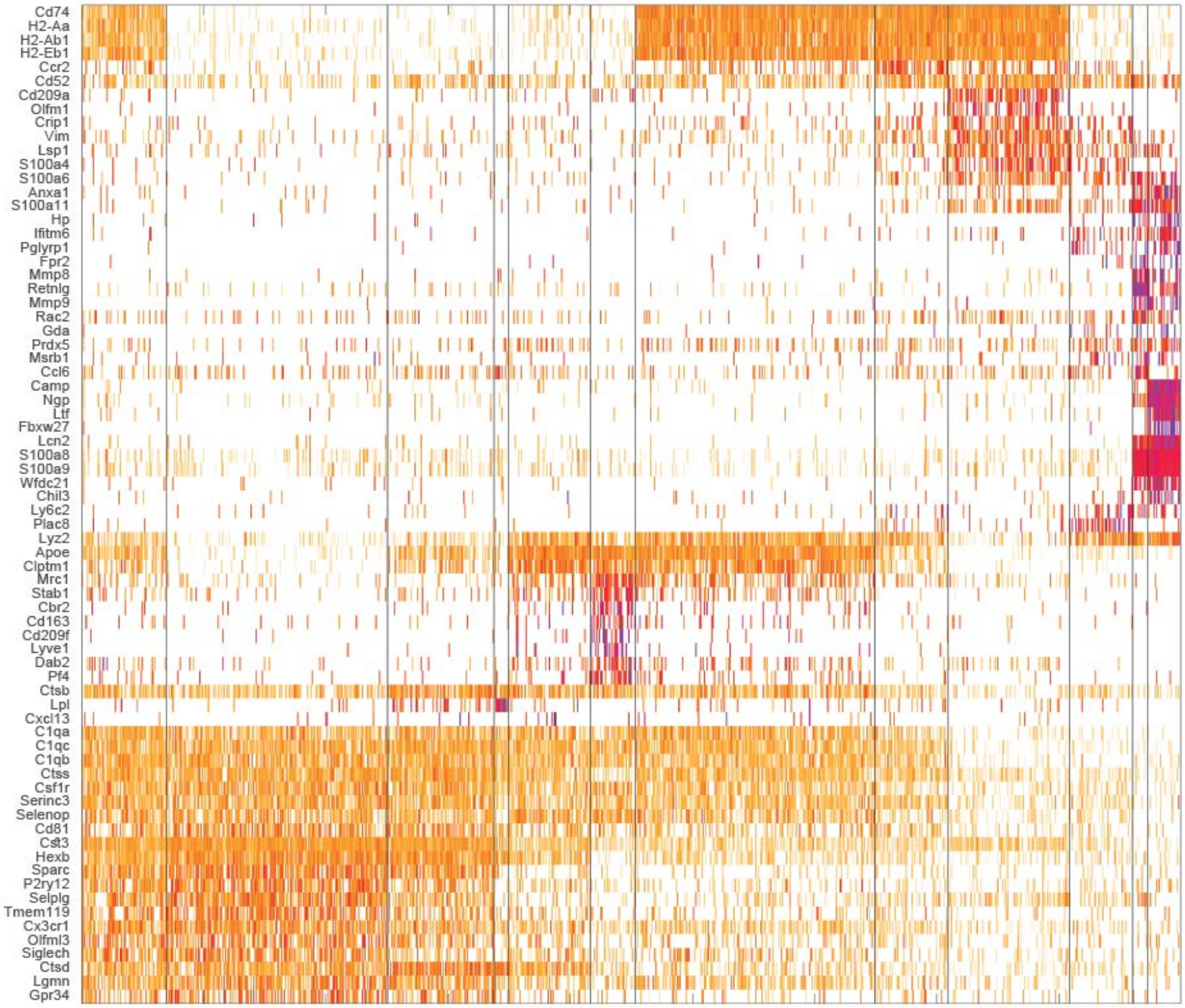

Supplementary Figure 8. Single cell RNA-seq reveals heterogeneous population of infiltrating monocytes. Heatmap showing the most differentially expressed genes in clustering of 899 cells into 12 clusters (metacells). Colors represent normalized log2 UMI count per gene.
